# Plasmonic nanophotothermal therapy: Destruction of 500 mm^3^ subcutaneous human basal cell carcinoma with gold nanoparticles and near infrared laser

**DOI:** 10.1111/srt.13890

**Published:** 2024-08-03

**Authors:** Sabrina Pesnel, Antoine Bertolotti, Sébastien Duquenne, Hassan Zahouani, Laurent Mortier, Jean‐Luc Perrot, Anne‐Laure Morel

**Affiliations:** ^1^ TORSKAL Sainte‐Clotilde Réunion France; ^2^ CICEC‐INSERM1410, Service de Maladies‐Infectieuses et Dermatologie CHU Réunion Saint‐Pierre Cedex Réunion France; ^3^ SELARL de pathologie Saint‐Pierre Cedex Réunion France; ^4^ Ecole Centrale de Lyon, LTDS – TMM Ecully Cedex France; ^5^ CHRU Lille, Service de Dermatologie, Hôpital Huriez Lille France; ^6^ CHU Saint Etienne, Service de dermatologie, Hôpital Nord Lille France

**Keywords:** basal cell carcinoma, gold nanoparticles, hyperthermia, NIR Laser, phototherapy

## Abstract

**Significance:**

Multilesional basal cell carcinoma (BCC) are spread on sun exposed skin areas, including arms, face and back. The first‐line treatment remains the surgical resection or Mohs surgery. Despite its high complexity, Mohs surgery is well practiced in USA and Germany and presents very good results both in esthetic and in carcinology point of view.

Large lesions more than 2 cm remain challenging to remove by topical cream used in photodynamic therapy (PDT). If these larger lesions are not treated in less than 1 month, they could grow deeply in the skin, thus enhancing the risk of reoccurrence and the severity of the disease. Despite this model herein studied, that is non melanoma skin cancer is a good prognostic cancer, the therapy aims to be applied to more aggressive melanoma skin cancers.

**Aim:**

Total regression of large cutaneous lesions less than 1 month with no reoccurrence.

**Approach:**

Tumor induction on murine model bearing a 500 mm^3^ subcutaneous lesion. Increasing dose of gold nanoparticles at fixed initial concentration C0 = 0.3 mg/mL, infused into the tumor then exposition of the region of interest to NIR medical laser to assess the therapy. One or two intratumoral administration(s) were compared to surgery and control, that is no treatment, laser alone or nanoparticles alone.

**Results:**

Gold nanoparticles alone or the NIR laser alone did not induce the tumor regression. The combination of laser and nanoparticles called plasmonic nanophotothermal therapy induced apoptosis. Derma and hypoderm do not show any visible gold nanoparticles and demonstrated a good cicatrization process.

**Conclusion:**

Plasmonic nanophotothermal therapy using two doses of gold nanoparticles was the only protocol that proved its efficacy on large lesions in 14 days, that is 500 mm^3^ on a murine model bearing human basal cell carcinoma.

## 1 INTRODUCTION

Untreated BCC can cause the spread of the malignancy on surrounding tissues.^1^ Excision is still recommended as a therapy of choice in the guidelines. The nonresectable areas and non‐eligible cases to radiotherapy could become locally advanced, metastatic cancers. The disgraceful scars and reoccurring tumors are usually treated by surgical excision, microscopically controlled surgery with 3D evaluation of excision margins,^2^ or Mohs surgery. These techniques require a high expertise and are time consuming. The literature describes that MCS has led to a reduction of the recurrence rate for primary tumors from 3%−4.1% to 2%−2.5% and for recurrent tumors from 3%−12.1% to 0%−2.4%.^3^ ^,^ ^4^

Systemic therapies are indicated for high‐risk BCC not amenable to radiotherapy or either surgery. Hedgehog pathway Inhibitors are approved as an alternative.

Photodynamic therapy (PDT) induced by protoporphyrin IX are widely used as skin cancer protocol. The mechanism of action of PDT well described reminds that ROS induce cytotoxicity.^5^ ^,^ ^6^ Recent methods suggest the delivery of drug in combination with PDT, more deeply with other physical techniques.^7^ ^–^ ^9^

In the management of high‐risk tumor, superficial radiotherapy is an alternative for thicker lesions up to 6 mm in depth.^10^ ^,^ ^11^

Nanocarriers have been widely described in preclinical assays as cancer drug delivery in clinic. The main cause of failure of these nanocarriers in translational research is the biodistribution parameters^12^ because of nonspecific targeting, toxic side effect due to the dissociation of toxic compounds in the body. Several attempts of physico‐chemical engineering of nanoparticles were studied. The main criteria are the specifications of the nano formulation: size, shape, surface charge, coating to provide safety, efficacy.

Noble metal nanoparticles present unique optical properties thanks to the interaction with incident light. These properties are called plasmonic.^13^ The localized surface plasmon resonance (LSPR) phenomena observed in noble metal nanoparticles are due to the resonance frequency in the visible and near infrared wavelength.^14^

Gold nanoparticles represent a good alternative to induce a thermal ablation of the tumor by stimulating the plasmon at the surface of nanoparticles,^15^ without any scars and with a quick response. The heat generated demonstrated good results on melanoma^16^ ^,^ ^17^ and non melanoma^18^ skin cancers in a preclinical level on animals. Moreover, they have showed good safety profile in preclinical and clinical stages.^19^ ^–^ ^21^ In this article, we have studied the use of spherical gold nanoparticles coated with glucose stimulated by near infrared laser in the thermal destruction of ticker lesions of BCC of 500 mm^3^ size on preclinical murine model. We have compared the results with those obtained on surgical excision by monitoring the tension index on skin. This showed an interesting profile of skin cicatrization by using few doses of nanoparticles within 14 days.

## 2 MATERIALS AND METHODS

### 2.1 Reagents and chemicals

Tetrachloroauric acid (HAuCl_4_), G‐glucose were purchased from Sigma‐Aldrich (Saint‐Quentin Fallavier, France). Hubertia ambavilla plant extract (TSK1) was sourced by selected crops in Reunion Island.

#### 2.1.1 Synthesis of gold nanoparticles (AuNP)

The gold nanoparticles were synthesized using the method previously described. An aliquot of AuNP solution was mixed with the same volume of D‐glucose under vigorous stirring for 1 h at room temperature. The solution was centrifuged and redispersed in 0.9% NaCl.

#### 2.1.2 Cell culture

The cell line TE 354.T was used. The human basal carcinoma cell line was purchased from ATCC (ATCC CRL‐7762) and cultured in DMEM supplemented with 10% heat‐inactivated fetal bovine serum, 50 U/mL penicillin, and 50 µg/mL streptomycin. The cell line was maintained at 37°C in 5% CO_2_ and 95% air in a humidified atmosphere.

### 2.2 In vivo assessment of the plasmonic photothermal activity of AuNP

#### 2.2.1 Animals and tumor model

BALB/c nude female mice, 7 weeks old were used. Animals were housed in plastic cages inside a controlled ventilated rack with free access to water and food. All experiments were performed in accordance with national animal care guidelines (EC directive 86/609/CEE, French decree no. 87‐848). Tumor xenografts were achieved by subcutaneous injection of tumor cells suspension (1 × 10^6^ cells in 100 µL NaCl 0.9%) into the right flank. To follow tumor growth, tumor volume (V in mm^3^) was measured with a caliper and was calculated as V = (length x width x thickness)/2.

#### 2.2.2 In vivo plasmonic photothermal treatment (PPTT)

##### Photothermal therapy (PTT)

**FIGURE 1:**
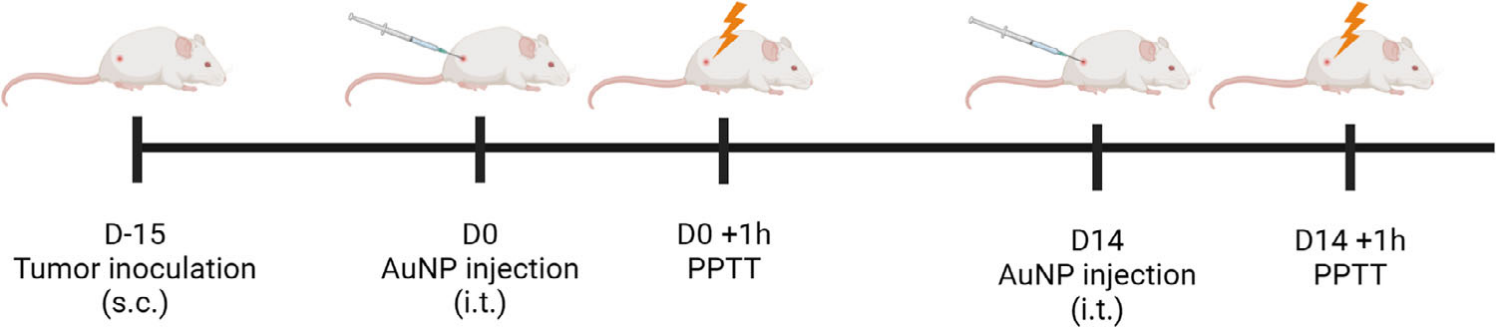
Protocol of administration of the plasmonic photothermal therapy (PPTT).

Mice were anesthetized with 2.5% isoflurane and a subcutaneous injection of buprenorphine (0.1 mg/kg) was done prior the injection of nanoparticles. Then, tumors were measured, and AuNP@G were injected in the center of the tumor with an insulin syringe 31G. One hour later, mice were placed under the medical laser (Hyper diode 808, Hyper Photonics, Italy) for treatment (Figure 1).

When tumors reached a volume of 500 mm^3 ^± 21% (D0), mice were randomly assigned into nine groups (four mice per group) as described in the Table 1. In the Ctrl group, mice were not treated; in the Laser group, NaCl 0.9% (injected volume = 20% of tumor volume) was injected into the tumor then the anesthetized mice received PPTT; in the AuNPs group, there were two different cohorts: one dose AuNP (injected volume = 20% of tumor volume or 40% at D0, and two doses AuNP (injected volume = 20% at D0 and D14); OD = 6 and OD = 12 corresponding, respectively, to 0.3 and 0.6 mg/mL) was injected into the tumor then the anesthetized mice received PPTT. Tumor irradiation was performed 1 h after injection of NaCl 0.9% or AuNP.

**TABLE 1:** Groups of mice for the in vivo assessment of the plasmonic photothermal activity.

Study	Group		Dose of AuNP@G (OD)	Administration route	Power of the laser (W/cm^2^)
Antitumor effect	1	Control	–		–
	2	Control laser	–	Into the tumor	1
	3	Control NP	12		–
	4	PTT 0.3 mg/mL 40%	6		1
	5	PTT 0.6 mg/mL 40%	12		1
	6	PTT 0.3 mg/mL 20%	6		1
	7	PTT 0.6 mg/mL 20%	12		1
	8	PTT 0.3 mg/mL 20% 2 treatments	6		1
Comparison with treatment of reference	9	Surgery	–		–

The treatment is described in the Table 2.

**TABLE 2:** Details of the treatment applied to the groups of mice.

	First treatment				Second treatment			
Group	Day	Volume of NP (% of tumor volume)	Power laser (W/cm^2^)	Laser probe (mm)	Day	Volume of NP (% of tumor volume)	Power laser (W/cm^2^)	Laser probe (mm)
2	0	–	1	25	14	–	1	25
4	0	40	1	25				
5	0	40	1	25				
6	0	20	1	25				
7	0	20	1	25				
8	0	20	1	25	14	20	1	25

For antitumor effect assessment, mice were weighed and tumors were measured every 3 days until the mice died or the tumor volume reached 1500 mm^3^ or the weight loss was upper than 20% of the initial weight.

Photothermal treatment consisted in a laser irradiation of the tumor, with the following settings:

1. Wavelength: 808 nm
2. Power: 1 W/cm^2^ (5 W with the 25 mm probe)
3. Duration: 10 min
4. Irradiated surface: 4,9 cm^2^ (diameter of the probe: 2.5 cm)

#### 2.2.3 Surgery: tumor resection

At day 0, some mice underwent tumor resection with a margin of 3 mm and skin was sutured

#### 2.2.4 Evaluation of toxic‐side effects

Maximum weight losses or gains, expressed as a percentage of the initial body weight of the experimental animals, was used to provide an assessment of the toxicity of AuNP@G. According to NCI (National Cancer Institute) criteria, a dose is considered toxic if the induced body weight loss is higher than 20% of the initial mouse body weight. It should be remembered that these weights also include the weight of the tumors, which increases with time.

#### 2.2.5 Evaluation of antitumor activity/tumor growth

Treatment efficacy was assessed in terms of the compound's effects on tumor volume for PTT‐treated mice relative to control vehicle‐treated mice.

Two evaluation criteria were used in parallel: (i) *Growth inhibition*, calculated as the ratio of the median tumor volume of AuNP@G‐treated versus control groups: T/C, % = (median tumor volume of AuNP@G‐treated group on day X/median tumor volume of control group on day X) x 100, the optimal value, being the minimal T/C ratio, which reflects the maximal tumor growth inhibition achieved; (ii) *Relative area under the tumor growth curve*, rAUC (%), representative of the tumor growth curve as a whole, reflects the overall effect of a test compound over time. rAUC =  [(area under the tumor volume growth curve of the treated group/median area under the tumor volume growth curve of the control group) x 100]. The more active the compound, the lower the rAUC value.

Tumor volume was monitored by measuring the tumors with a digital caliper and according to the formula (L x l x e)/2.

Histological analyses were performed at different time points after treatment (21 days, 3 months, and 6 months) on mice with complete regression and on operated mice to confirm the absence of tumor.

#### 2.2.6 Comparison with surgery (treatment of reference)‐ Tension index measurement

Skin prints were taken using Silflo resin to analyze the skin microrelief and study the impact of the treatments (PPTT and surgery) on the skin.

#### 2.2.7 Statistical analysis

Data were presented as mean ± STD. Statistical analysis was performed with GraphPad 8.0.

## 3 RESULTS

**FIGURE 2:**
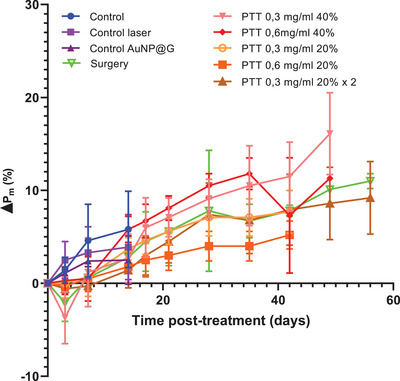
Effects of AuNP@G administered into the tumor and laser irradiation on the body weight of mice bearing subcutaneous human BCC xenograft.

Treated BCC‐bearing mice did not show any significant weight loss (the toxicity limit set by the NCI is −20%) (Figure 2) and no clinical signs of toxicity was observed except in the groups 4 and 5 which corresponds to 40% TV of NP injection.

Indeed, in this group, important weight loss was observed, and necropsies showed burns in the intestines. If some contradictions still exist on the greater efficiency of one or the other method in vitro,^22^ ^,^ ^23^ all the studies seem to agree on the fact that PTT will induce more apoptosis if continuous irradiation is used and conversely will induce more necrosis in the case of pulsed irradiation.^24^ However, necrosis leads to significant local destructive inflammation, thus creating an inflammatory microenvironment conducive to the development of potential surviving cancer cells.^25^

**FIGURE 3:**
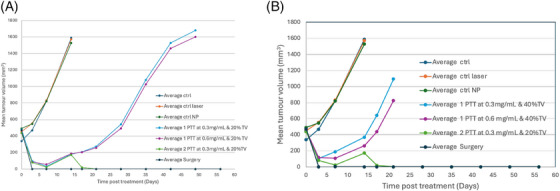
Efficacy treatment of TSK01 after one or two injections of nanoparticles followed by NIR laser (A) with 20%TV of injected nanoparticles or (B) with 40% TV of injected nanoparticles.

Figure 3 and Table 3 show the curves of the monitoring of tumor size by varying either the concentration, or the volume to be injected.

**TABLE 3:** Monitoring of the weight loss during the treatment.

Compound	Group	Maximal median body weight change^a^ (%)	Body weight loss > 20% (%)	Presumed drug related deaths^b^ (%)
AuNP@G	4	−20.2	20	20
	5	−21.4	20	20
	6	−2.6	0	0
	7	−1.2	0	0
	8	−1.3	0	0

^a^ Body weight changes are gains or losses expressed as a percentage of the initial body weight. At each day of weighing, the median value of body weight changes is determined for each experimental group. Then the maximal body weight loss recorded over time is defined. According to NCI criteria, a dose is considered highly toxic if the induced body weight loss is greater than 20% of the initial body weight

^b^ Death is presumed to be drug‐related if the treated animal died before the control animals. According to NCI, a dose is considered as toxic if the percentage of toxic deaths is higher than 20%

We can observe a regression of the tumor from 1 to 14 days post injection, then an increase of the tumor volume at 14 days. Twenty percent of nanoparticles injection (Figure 3A) is not sufficient to induce a complete regression of the tumor but demonstrates no toxicity, whereas 40% of injection in Figure 3B seems to be toxic.

**FIGURE 4:**
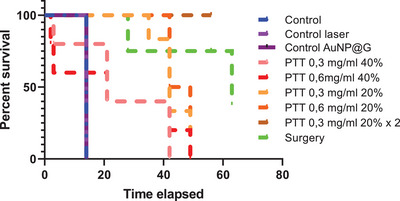
Survival curve.

Figure 4 is the survival curve indicating the highest rate for surgery group, followed by two doses of nanoparticles and 20%TV for one injection.

**FIGURE 5:**
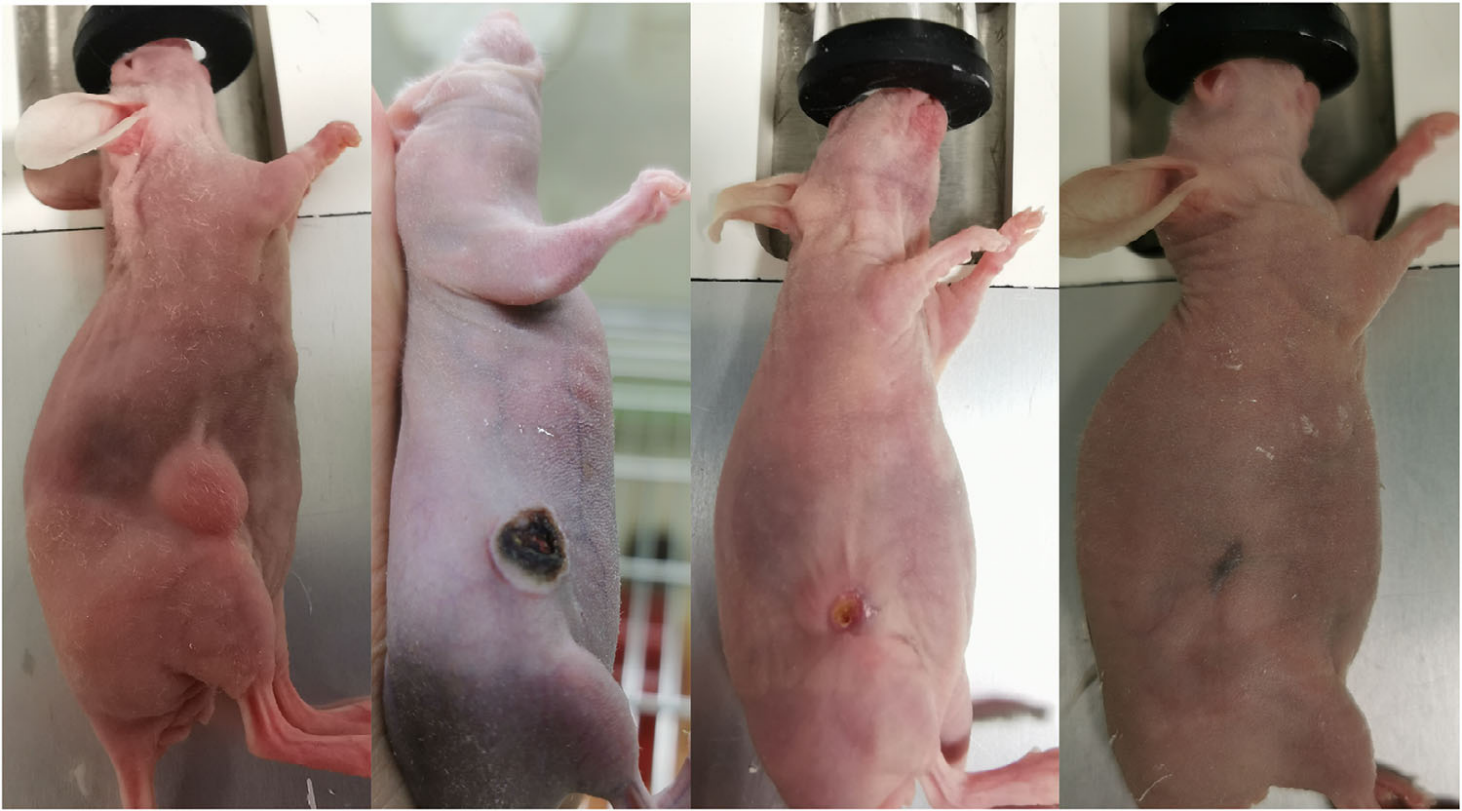
Effects of PTT on the tumor volume of a mouse bearing subcutaneous human BCC xenograft before treatment and 3, 14, and 21 days after treatment.

Figure 5 showed the tumor evolution on a PTT‐treated mouse.

**FIGURE 6:**
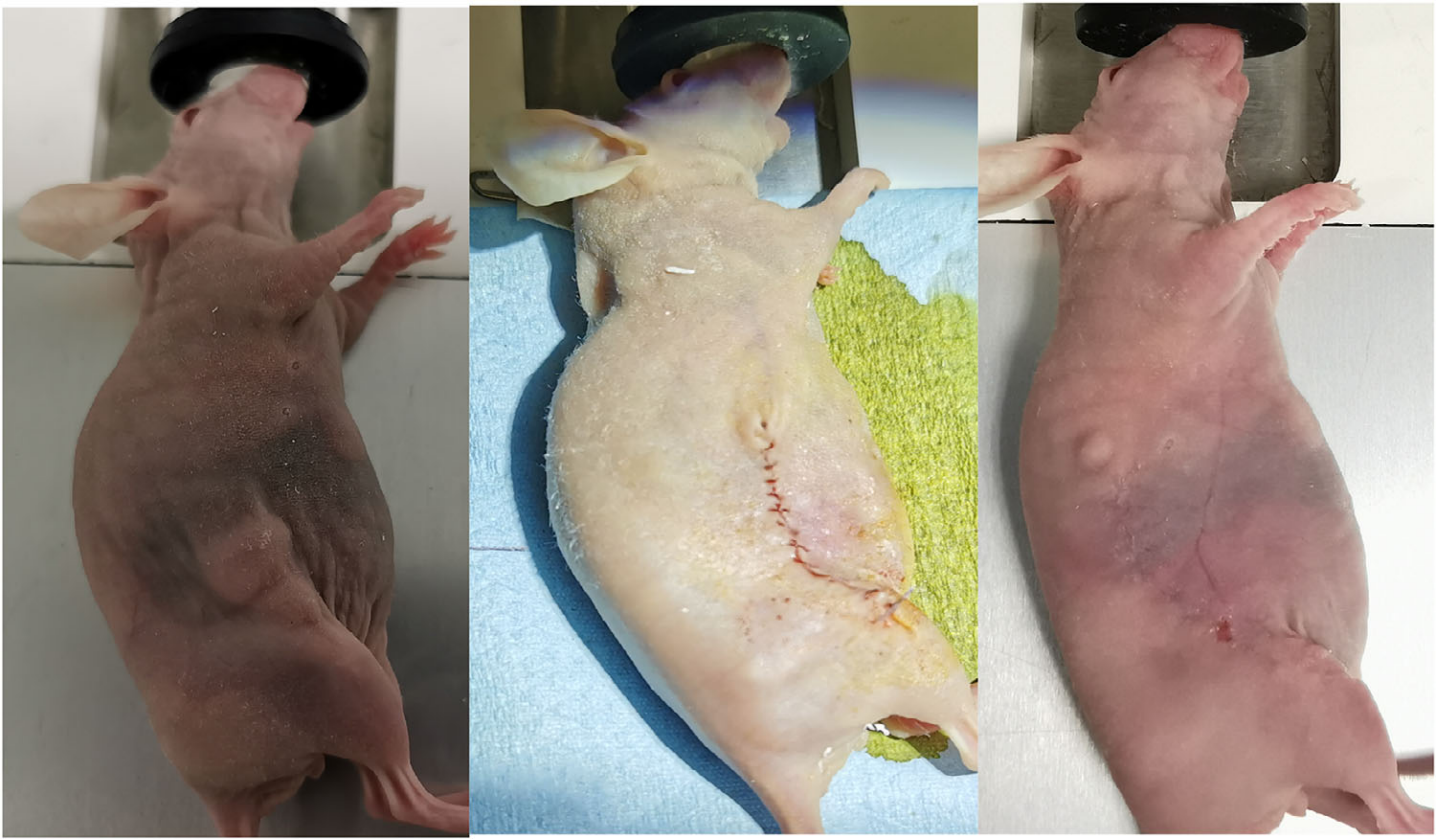
Pictures of a mouse bearing subcutaneous human BCC xenograft before surgery and just after and 21 days after surgery.

Figure 6 showed the evolution of the scar after surgery. Two mice treated by surgery showed a tumor recovery accompanied by ascites.

**FIGURE 7:**
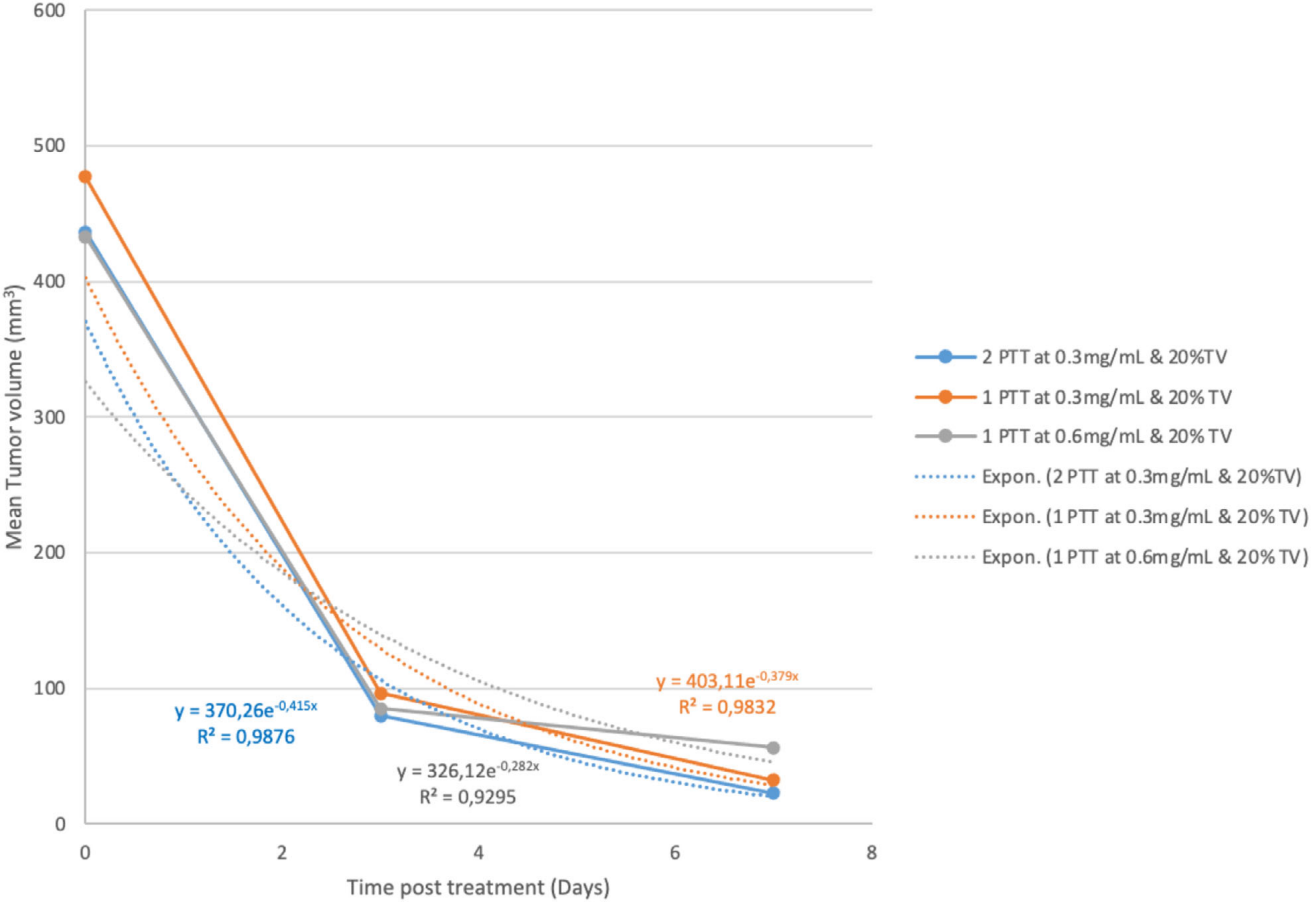
Regression of the tumor volume during the first injection of nanoparticles at 20% of TV injection.

Figure 7 demonstrates the regression of the tumor volume during the first injection at 20% of TV.

We observed that the exponential curves are quite similar for the injection of 20% of gold nanoparticles for both concentration 0.3 and 0.6 mg/mL. The curves at 40% TV are not compared because of significant toxicity.

**FIGURE 8:**
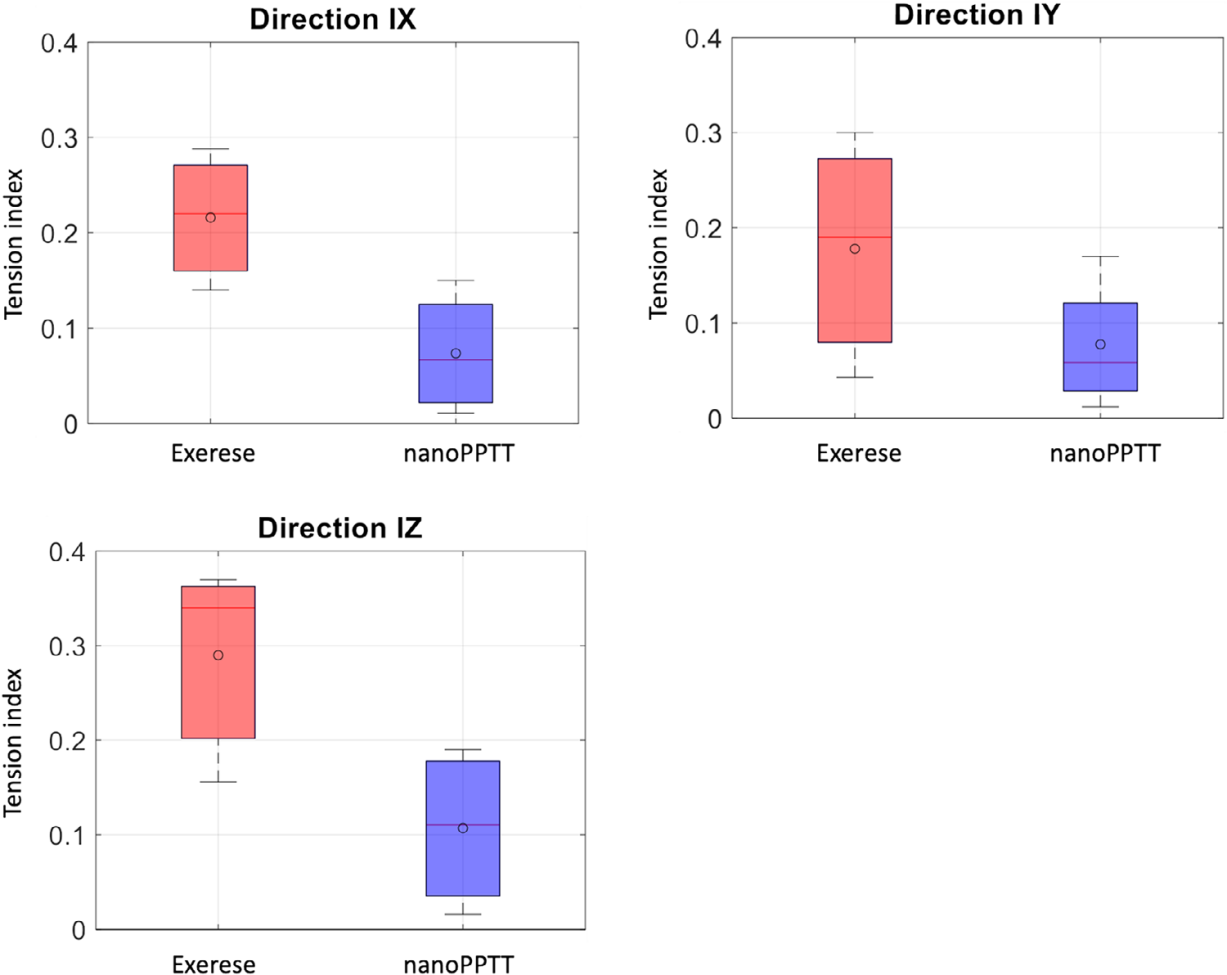
Tension index measured using Silflo resin to analyze the skin microrelief and study the impact of the treatments on the skin.

Figure 8 shows the tension index measured using Silflo resin to analyze the skin microrelief. We can see that the treated mice have less tension on their skin on X, Y, and Z axis than the surgery group.

## 4 DISCUSSION

We previously demonstrated partial regression on melanoma murine model with one injection on primary site, then the complete regression of small lesions (50 mm^3^) of BCC with two doses of nanoparticles at 20% TV within 3 days instead of 14 days between two injections. In small lesions, the more rapidly we interrupted the tumor proliferation by reducing the delay between two injections, the better was the chance to have a complete regression.

As the eradication of the tumor is provided by a correct ratio of concentration of gold in the tumor and power density, it is fundamental to better understand the pharmacokinetic of these coated nanoparticles, which will allow us to monitor absorption, thus the concentration of gold in the tumor, the distribution, and metabolization of these nanoparticles. The plasmonic nanophotothermal therapy is a mechanism of action depending on these parameters for the optimization of the physical destruction of the tumor by heat.

A significant antitumor activity was observed after each injection of nanoparticles and that was not correlated to the volume (20% or 40% of the tumor volume). In this study, no group was retreated 3 days after the first injection limited by the presence scabs on the tumors. The second injection performed on group 8 was the only protocol demonstrating a complete regression in 14 days.

The burns in the intestines leading to mortality at 40% of TV demonstrates the correlation between the gold quantity accumulated in the tumor submitted to near Infrared stimulation and the hyperthermia generated in the tissue. The high injected volume induced “pockets” of nanoparticles around the tumor and the irradiation caused a high hyperthermia leading to serious adverse events.

Although pulsed irradiation allows better temperature control than continuous irradiation, the literature indicates that the size of the tumor treated by this method will be extremely limited, making it impossible to envisage the total destruction of a primary tumour.^26^ ^–^ ^29^ In addition, it has been shown that the pulsed irradiation of gold nanoparticles leads to a modification and degradation of these, impacting its photothermal capacities. Thus, following a first irradiation, the possibility of carrying out a second irradiation no longer exists and the toxicity profile of these “modified nanoparticles” is not known.^27^ ^,^ ^28^

To confirm the validity of our choice, we were interested in the results of clinical trials yet described. Nanospectra in the treatment of cancerous lesions on the prostate by PTT used hybrid silica‐gold nanoparticles. Nanoparticles were excited by continuous irradiation and no side effects relating to the irradiation procedure were noted.^30^ ^,^ ^31^

The objective of our treatment is to cause the death of cancer cells by PTT and not by intense laser power, which would then cause the death of healthy cells as well and therefore, undesirable side effects. Thus, various clinical trials were reported that studied a treatment by PTT, via an excitation of nanoparticles by a continuous NIR laser and having caused no side effects relating to the irradiation procedure. 3.0–5.0 W for 3–4 min^30^ or 4.5–6.5 W for 3 min^31^ were applied to the prostate tumor and 35–44 W/cm^2^ for 7 min^32^ on atherosclerosis.

Figure 7 indicates that the curves is a double exponential and quite similar with 20% or 40% of tumor volume injection of nanoparticles. Considering these parameters, we have selected the 20% of tumor volume injection dose of nanoparticles to reach a complete tumor regression.

Figure 8 shows the tension index in three directions IX, IY, and IZ measured using Silflo resin to analyze the microrelief of the skin after nanoPPTT or after the exercise. A reduced tension index on nanoPPTT is observed versus surgical resection, especially on IZ direction.

## 5 CONCLUSION

We have demonstrated a treatment consisting in injecting two doses of nanoparticles followed by near infrared irradiation for the thermal ablation of 500 mm^3^ tumors on skin. We observed apoptosis causing complete regression of the tumors within 14 days. The comparator (surgery) showed significant change of skin tension of mice suggesting an improvement of the cicatrization of the wound induced by the thermal ablation. We should remain cautious regarding the ADME parameters, especially the quantity of gold in the tumor. Our comprehension of the kinetic of internalization of the nanoparticles helped us to design the correct clinical synopsis for future clinical investigation in Man.

Beyond this esthetic requirement which goal are widespread, and the high performance of the thermal ablation on preclinical level, we pave the road on the use of gold nanoparticles in dermatology as medical imaging agent, called nanotheranostic.

## CONFLICT OF INTEREST STATEMENT

The author declare no conflict of interest.

## ETHICS STATEMENT

Animal experiments were performed in accordance with national animal care guidelines (EC directive 86/609/CEE, French decree no. 87‐848).

## Data Availability

The data supporting this article's findings are available upon reasonable request from the corresponding author.
